# Ultrastructural Changes and Antitumor Effects of Doxorubicin/Thymoquinone-Loaded CaCO_3_ Nanoparticles on Breast Cancer Cell Line

**DOI:** 10.3389/fonc.2019.00599

**Published:** 2019-07-03

**Authors:** Kehinde Muibat Ibiyeye, Norshariza Nordin, Mokrish Ajat, Abu Bakar Zakaria Zuki

**Affiliations:** ^1^Laboratory of Molecular Biomedicine, Institute of Bioscience, Universiti Putra Malaysia, Seri Kembangan, Malaysia; ^2^Genetics and Regenerative Medicine Research Centre, Faculty of Medicine and Health Sciences, Universiti Putra Malaysia, Seri Kembangan, Malaysia; ^3^Department of Veterinary Preclinical Sciences, Faculty of Veterinary Medicine, Universiti Putra Malaysia, Seri Kembangan, Malaysia

**Keywords:** doxorubicin, thymoquinone, CaCO_3_ nanoparticle, breast cancer, combination therapy

## Abstract

**Background:** Combination chemotherapy of anticancer drugs is extensively being researched since it could reduce multidrug resistance and side effects as a result of lower dosage of each drug. In this study, we evaluated the effects of doxorubicin-loaded (Dox-ACNP), thymoquinone-loaded (TQ-ACNP) and a combined doxorubicin/thymoquinone-loaded cockle shell-derived aragonite calcium carbonate nanoparticles (Dox/TQ-ACNP) on breast cancer cell line and compared with their free drugs counterpart.

**Methods:** Cell viability using MTT assay, apoptosis with Annexin V-PI kit, morphological changes using contrast light microscope, scanning electron microscope and transmission electron microscope, cell cycle analysis, invasion assay, and scratch assay were carried out. The cell viability was evaluated in breast cancer cell line (MDA MB231), normal breast cells (MDF10A) and normal fibroblast (3T3).

**Results:** MDA MB231 IC_50_ dosages of drug-loaded nanoparticle were not toxic to the normal cells. The combination therapy showed enhanced apoptosis, reduction in cellular migration and invasion when compared to the single drug-loaded nanoparticle and the free drugs. Scanning electron microscope showed presence of cell shrinkage, cell membrane blebbing, while transmission electron microscope showed nuclear fragmentation, disruption of cell membrane, apoptotic bodies, and disruption of mitochondrial cistern.

**Conclusion:** The results from this study showed that the combined drug-loaded cockle shell-derived aragonite calcium carbonate nanoparticles (Dox/TQ-ACNP) showed higher efficacy in breast cancer cells at lower dose of doxorubicin and thymoquinone.

## Introduction

In the last few decades, combination therapy has been more reliable than mono-therapies because cancer cells possess survival multiple pathways including uncontrolled proliferation, evasion of apoptosis, stimulation of angiogenesis and propensity for local invasion and distance metastases (1). The limitations concerning single-drug systemic administration include drug resistance, rapid renal clearance and poor bioavailability. Also, accumulation of drug at the tumor sites is usually too low thereby requiring a higher drug dose which may causes severe adverse side effects and may not suppress cancer cell growth as a result of the heterogeneous distribution of cells within the tumor (2). Combination chemotherapy comprising of two or more anticancer drugs has been shown to reduce drug resistance and side effects due to lower dosage of each drug (3). However, to obtain significant anti-tumor effect in combination therapy with reduced normal tissue toxicity due to the drugs' different physical and chemical properties, is still a challenge.

Nanotechnology has shown a great advantage in drug delivery for cancer treatment by enhancing buildup of cytotoxics in tumor tissue, specificity in tumor targeting, reducing the cytotoxics side effect on normal cells, reducing systemic side effect, increasing drug solubility, and increasing maximum tolerated dose (3, 4). Interestingly, Calcium carbonate (CaCO_3_) nanoparticles has got immense attention because it is the most biocompatible; this makes aragonite an excellent biological drug delivery systems of anticancer drugs (5, 6). Calcium carbonate microparticles have been stated to cause growth stasis due to increase pH in the cancer tissue (7).

Doxorubicin (Dox) is a commonly used cytotoxic drug in the treatment of breast, leukemia, and other types of cancer. Dox acts by inhibiting enzyme topoisomerase II. Topoisomerase I and II alter DNA topography through DNA strand cleavage, strand passage and relegation (8).

Thymoquinone (TQ) is a major active constituent of black seeds (Nigella sativa). The seeds have been used to treat a range of ailments in traditional medicines (9). TQ has been shown to have antineoplastic effects in both *in vitro* and *in vivo* studies (10–12). TQ sensitizes cancer cells toward radiotherapy, chemotherapy and/or immunotherapy and reduces therapy-related side effects in normal cells. Thymoquinone enhanced the cytotoxic properties of ionizing radiation (13) and doxorubicin in multi-drug resistant variant of MCF-7 cells, paclitaxel and resveratrol (13–15).

The goal of this study was to evaluate the anticancer effects of doxorubicin-loaded (Dox-ACNP), thymoquinone-loaded (TQ-ACNP) and combined doxorubicin/thymoquinone-loaded cockle shell-derived aragonite CaCO_3_ nanoparticles (Dox/TQ-ACNP) compared with their free drugs counterpart on breast cancer cell line.

## Materials and Methods

### Preparation of ACNP and Drug Loading

The preparation of ACNPs, drug loading and characterization of Dox-ACNP, TQ-ACNP, and Dox/TQ-ACNP were carried out in accordance with Ibiyeye et al. (16).

### Cell Lines

MDA-MB-231 and 3T3 cell line (ATCC) were maintained in DMEM: F12 (Gibco) with 10% fetal bovine serum (Tico Europe), 1% antibiotics, and 10% FBS. MCF-10A cell was cultured in DMEM-F12 media with 0.5 μg/ml hydrocortisone, 10 μg/ml insulin, 20 ng/ml hEGF, and 10% FBS. All cells were incubated in 5% CO_2_ at 37°C. Cells at 80–90% confluence was used for experiment.

### Cell Viability Assay

The cytotoxic effect of drug loaded ACNPs was assessed with MTT reagent (Nacalai Tesque, Japan). In this assay live cells reduce the yellow MTT reagent, to purple formazan crystals and it is then quantified. Briefly, MDA-MB-231 cell line were cultured with different concentrations of drug-loaded ACNP and free drugs. Cells were seeded (5 × 10^3^ cells/well) in a 96-well plate then incubated overnight. The media was removed, then 200 ul of complete media containing different concentration of drug (ranging from 0 to 10 μg/ml) was added. For the non-neoplastic cells, MCF-10A and 3T3 cell lines were cultured with different concentrations of Dox-ACNP, TQ-ACNP and Dox/TQ-ACNP (ranging from 0 to 50 μg/ml).

Cell were then incubated for 24, 48, and 72 h. After appropriate treatment, 20 μl MTT solution (5 mg/ml) was added into each well and incubated at 37°C for 4 h. The media was then removed by pipetting, and the formazan crystals formed were dissolved with 200 μl DMSO. The absorbance of each well was read at 570 nm by a microplate reader (Tecan Infinite, Mannedorf, Switzerland). The concentration of treatment that has 50% inhibition (IC_50_) was used for further studies (17).

### Combination Index (CI)

The CI was calculated using CompSyn software, to evaluate the synergism between the two drugs using classic isobologram equation of Chou-Talalay. CI>1.3 antagonism; CI 1.1–1.3 moderate antagonism; CI 0.9–1.1 additive effect; CI 0.8–0.9 slight synergism; CI 0.4–0.8 synergism; CI 0.2–0.4 strong synergism (18).

### Safety Assessment of Drug-Loaded ACNP in Non-neoplastic Cells

MCF-10A and 3T3 cell lines were cultured with IC_50_ dosage of drug-loaded ACNP (Table 1) corresponding to MDA-MB-231 cells for 24, 48, and 72 h. The cells were then analyzed as above.

**Table 1:** Showing IC_50_ data of free and drug loaded ACNPs at 24, 48, and 72 h of treatment.

	**Dox (μg/ml)**	**Dox-ACNP (μg/ml)**	**TQ (μg/ml)**	**TQ-ACNP (μg/ml)**	**Dox/TQ (μg/ml)**	**Dox/TQ-ACNP (μg/ml)**
24 h	1.765 ± 0.096	2.588 ± 0.067	1.068 ± 0.038	0.8103 ± 0.145	1.601 ± 0.048	1.958 ± 0.183
48 h	0.3167 ± 0.192	0.974 ± 0.079	0.5591 ± 0.081	1.457 ± 0.143	0.552 ± 0.124	0.4656 ± 0.06
72 h	0.1453 ± 0.246	1.338 ± 0.126	0.3675 ± 0.09	1.44 ± 0.179	0.319 ± 0.5	0.09532 ± 0.379

### Morphology Assessment

#### Light Microscopy Imaging

For light microscopy imaging, MDA-MB-231 cells were seeded at a density of 1.5 × 10^5^ cells/well in 6-well plate and grown overnight at 37°C. For MDA-MB-231 the medium was then replaced with fresh medium containing different IC_50_ of the drug formulations and incubated for 24, 48, or 72 h, respectively.

#### Scanning Electron Microscope (SEM)

Surface morphology of MDA-MB-231 cells were observed using SEM. The cells were seeded at a density of 1.5 × 10^5^ cells/well in 6-well plate and grown overnight at 37°C. The medium was then replaced with fresh medium containing different drug formulations and incubated for 72 h at 37°C. Samples were then washed with PBS, fixed in 2.5% (v/v) glutaraldehyde and kept in 4°C for 4 h. Then washed with buffer three times and post-fixed with 2% osmium tetroxide for 2 h at 4°C, dehydrated through different concentrations of ethanol (35, 50, 70, 80, 85, and 95%) 10 min each and twice at 100%. Thereafter, immersed in acetone for 10 min. The cell samples were mounted onto an aluminum stub, point dried, sputtered gold coated (E5100 Polaron, UK) and they were examined under SEM (JOEL-64000, Japan). Surface and morphological changes in the cells are then observed and recorded (19).

#### Transmission Electron Microscopy (TEM)

For the analysis of the ultrastructure of treated MDA-MB-231, TEM was used. The sample were fixed, blocked, washed, dehydrated, embedded in resin, sectioned, stained, and viewed under a transmission electron microscope (Philips, Eindhoven, Netherlands) as previously described by Kamba et al. (19).

### Cell Cycle Analysis

The effect of treatment on cell cycle was examined using Propidium iodide (PI) flow cytometry kit (abcam USA) according to manufacturer's instructions. The DNA content is used to differentiate the cell cycle phase; where G1 phase is 2n, S phase is between 2n and 4n, and G2 phase is 4n. PI binding to the DNA is proportional to DNA content. Briefly, cells were seeded (1.5 × 10^5^ cells/well) in 6-well plates and incubated overnight. Fresh media containing different treatment was added and incubated for 24, 48, or 72 h at 37°C. After incubation period, the cells were detached and fixed with 70% ethanol for at least 2 h and stained with PI solution (containing RNase) in the dark for 20 min and then analyzed with a flow cytometer (CyAnTM Flowcytometry, USA).

### Annexin V Assay

The apoptotic effect of the drug-loaded ACNP and free drugs on breast cancer cells was assessed with Annexin V-PI kit (Nacalai Tesque, Japan). Annexin V can specifically bind to phosphatidylserine which diffuses from inner cell membrane to outer cell membrane during apoptosis. Briefly, cells were seeded (1.5 × 10^5^ cells/well) in a 6-well plates and incubated overnight. After appropriate treatment, the cells were detached with trypsin, washed, and incubated with Annexin V-FITC/PI solution (5 μl Annexin V and 5 μl PI) in the dark for 15 min. The cells were then analyzed with a flow cytometer (CyAnTM Flowcytometry, USA).

### Cell Migration Assay

The “wound-healing” assay was used to investigate the effect of drug-loaded ACNP and free drugs on breast cancer cells migration. The principal is to create a wound within cell culture and examine the cell migration and proliferation to fill the wound. Briefly, cells were seeded in 6-well plates and incubated at 37°C up to about 80–90% confluence. The wound was created using 200 μl pipette tip and rinsed with PBS to remove detached cells. Fresh media with appropriate treatment was added and incubated for 48 h. The observation of the cells was recorded at 0, 6, and 24 h with a light microscope.

The following formula was used to calculate the rate of migration (20):

$$\begin{array}{l} \text{Percentage of wound closure}=(\text{Area of wound at 0 hr -}\\ \text{ Area of wound at n hr/Area of }\\ \text{ wound at 0 hr})\;\times \text{ 100} \end{array}$$

### Invasion Assay

Using commercially available invasion assay kit (CHEMICON cell invasion assay kit, EMD Millipore), the invasiveness of cancer cells was investigated by examining the number of cells that had migrated through BME toward the chemo-attractant. The procedure was carried out according to the manufacturer's instructions. Briefly, 40 μl of basement membrane solution was added to coat desired well of top chamber and incubated for 1 h. Cells will be cultured to 80% confluence. Prior to assay, the media was replaced with serum free media to starve the cells for 18–24 h. The cells were then harvested and suspended at 1 × 10^6^ cells/ml. 200 μl of media containing 10–20% fetal bovine serum was added to the bottom chamber. FBS serve as the chemo-attractant. 50 μl of cell suspension was added to the upper chamber with the desired treatment and incubated for 48 h.

The percentage cell invasion will be calculated as follows (20):

$$\begin{array}{l} (\text{Number of cells in lower chamber}\\ \text{/ Total number of cells added to top chamber})\text{ x100} \end{array}$$

### Statistical Analysis

All statistical analysis was performed with Graphpad Prism version 7. The data were presented as statistical means ± S.E. The *p*-value < 0.05 was set to be significant. The comparison between groups was done using the one way or two way ANOVA with Tukey *post hoc* test.

## Results and Discussions

### Cell Viability

The cell viability studies were evaluated on MDA-MB-231 breast cancer cells using an MTT assay. We tested free Dox vs. Dox-ACNP, free TQ vs. TQ-ACNP and free Dox/TQ vs. Dox/TQ-ACNP (Dox: TQ = 3:2) by incubating them with the cells at 0–10 μg/ml for 24, 48, and 72 h.

**Figure 1:**
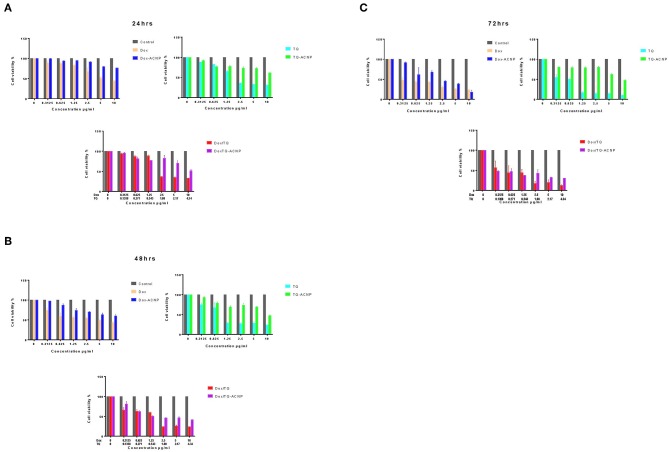
Percentage cell viability of MDA MB 231 after treatments with free and drug loaded ACNP for **(A)** 24 h **(B)** 48 h **(C)** 72 h, respectively.

As shown in Figures 1A–C, at all time period the cell viability of the free Dox, TQ and Dox/TQ was less than those of Dox-ACNP, TQ-ACNP, and Dox/TQ-ACNP, respectively. The cell viability progressively reduced has the treatment dose increased in a time dependent manner.

MDA-MB-231 cells are more sensitive to free drugs than the drug-loaded ACNP. This maybe because the free drug act on the cells before ACNP releases it content, making their onset of action slower than the free drugs. Dox/TQ-ACNP was the most effective in reducing the cell viability of the breast cancer cells when compared to Dox-ACNP and TQ-ACNP. This is due to the synergy between the loaded Dox and TQ (section IC_50_ and Combination Index) and also the enhanced cellular uptake and nuclear localization demonstrated by Dox/TQ-ACNP as compared with Dox-ACNP (16).

Fu et al. also noticed that the cell viability of Dox-loaded calcium carbonate nanoparticle was higher than free Dox in UMR-106 cells, rat osteogenic sarcoma cell line (21). Docetaxel-loaded cockle shell-derived calcium carbonate nanoparticles' cell viability in MCF 7 and 4T1 human and mice breast cancer cell line was also reported to be higher than free docetaxel (20). TQ-encapsulated chitosan nanoparticles was twice as potent as inducing cytotoxicity on MCF7 than free TQ (22). Paclitaxel/thymoquinone-loaded PLGA nanoparticle is more effective in reducing cell viability of MCF7 than free paclitaxel or paclitaxel-loaded PLGA nanoparticles alone (14). Soni et al. (14) also claimed that thymoquinone augmented the cytotoxic activity of paclitaxel. Doxorubicin and curcumin co-delivered by polymeric micelles nanoparticle showed increased cytotoxic or apoptotic activities on breast cancer cells, 4T1, compared with Dox-loaded or Cur-loaded at equal concentrations; this mainly attributed to improved cellular uptake of doxorubicin by curcumin (23).

### IC_50_ and Combination Index

IC_50_ (inhibitory concentration at 50% viability) was quantified from the cell viability data shown in Figures 1A–C using Graphpad Prism 7. As shown in Table 1, the IC_50_ of free Dox was significantly lower than Dox-ACNP at all time period. At 24 h, IC_50_ of TQ was higher than that of TQ-ACNP although not significant. At 48 and 72 h the IC_50_ of TQ-ACNP was significantly higher than TQ. There was no significant difference in the IC_50_ of Dox/TQ and Dox/TQ-ACNP at all time period, but IC_50_ of Dox/TQ was lower at 24 h while IC_50_ of Dox/TQ-ACNP was lower at 48 and 72 h.

Combination index (CI) along with results interpretations were calculated for Dox/TQ and Dox/TQ-ACNP at 24, 48, and 72 h of treatment (Table 2). The CI values for Dox/TQ-ACNP at 24, 48, and 72 h showed additive, synergy and strong synergy, respectively, while Dox/TQ showed moderate antagonism, slight synergy and antagonism, respectively. This result showed that Dox/TQ-ACNP is more effective in inhibiting the growth of breast cancer cells than free Dox/TQ combination despite the increased in cell viability noticed in Dox/TQ-ACNP at similar dose with DoxTQ (Figure 1C).

**Table 2:** CI and interpretation for the free Dox and TQ combination treatment and Dox/TQ-ACNP at 24, 48, and 72 h of treatment.

	**Dox/TQ**			**Dox/TQ-ACNP**		
	**IC_**50**_ (μg/ml)**	**CI**	**Interpretation**	**IC_**50**_ (μg/ml)**	**CI**	**Interpretation**
24 h	1.601/0.696	1.26	Moderate antagonism	1.958/0.851	0.97	Additive effect
48 h	0.552/0.24	0.8	Slight synergism	0.4656/0.202	0.5	Synergism
72 h	0.319/0.138	2.5	Antagonism	0.0953/0.041	0.13	Strong synergism

*Interpretation of results: CI > 1.3 antagonism, CI 1.1–1.3 moderate antagonism, CI 0.9–1.1 additive effect, CI 0.8–0.9 slight synergism, CI 0.6–0.8, moderate synergism, CI 0.4–0.6 synergism; CI 0.2–0.4 strong synergism*.

Curcumin/doxorubicin combination-loaded nanoparticle showed synergism against U87MG glioblastoma cells (CI of 0.73) (24). Similar finding was reported by Soni et al. (14) that the CI for the combination of paclitaxel/thymoquinone-loaded PLGA nanoparticle was 0.688. This indicates that paclitaxel/thymoquinone-loaded combination exhibits synergistic interaction (14). Thymoquinone has been demonstrated in many *in vivo* and *in vitro* studies to sensitizes cancer cells, including breast, ovarian, prostatic, colorectal cancers, leukemia, toward conventional radiotherapy, chemotherapy, and immunotherapy (13). The proposed mechanism include up-regulation of anti-tumorigenic proteins, suppression of pro-cancerous signaling proteins, increased induction of DNA damage and cell cycle arrest, and up regulation of pro-apoptotic proteins etc.

The doses of Dox-ACNP and Dox at 2.1 μg/ml, TQ-ACNP and TQ at 0.9 μg/ml, Dox/TQ-ACNP and Dox/TQ at 1.8 μg/ml for 24 h treatment; Dox-ACNP and Dox at 0.6 μg/ml, TQ-ACNP and TQ at 1 μg/ml, Dox/TQ-ACNP and Dox/TQ at 0.5 μg/ml for 48 h treatment; Dox-ACNP and Dox at 0.7 μg/ml, TQ-ACNP and TQ at 0.9 μg/ml, Dox/TQ-ACNP and Dox/TQ at 0.2 μg/ml for 72 h treatments were used for further experiment to compare the free and drug-loaded ACNP counterpart; i.e., the average of the IC_50_ value of the free and corresponding drug-loaded ACNP for each treatment hrs.

### Cell Viability Assay of Drug-Loaded ACNP in Non-neoplastic Cells

**Figure 2:**
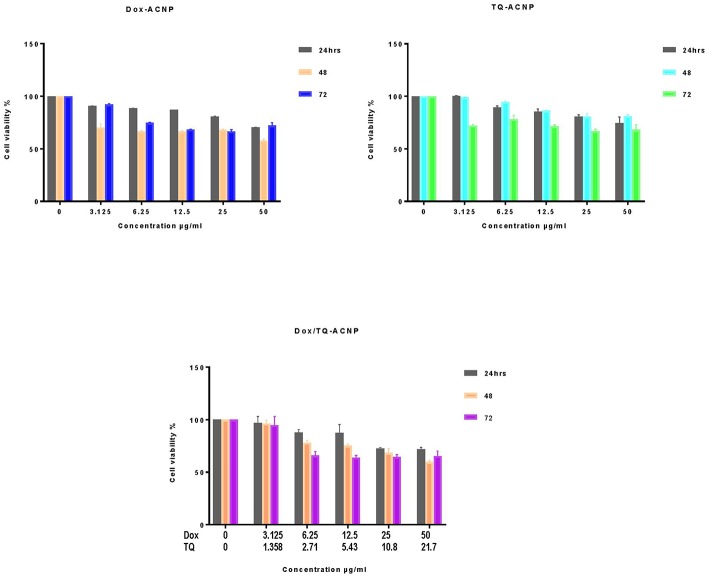
Percentage cell viability of 3T3 cells after treatments with drug loaded ACNP at concentration ranging from 0 to 50 μg/ml for 24, 48, and 72 h.

The cell viability studies were evaluated on 3T3 and MCF-10A cells using an MTT assay. As shown in Figure 2, for Dox-ACNP, at 24 h the cell viability of 3T3 slightly decreased from 90.7 to 70.4% at treatment dose of 3.125 and 50 μg/ml, respectively. 3T3 cell viability dropped to 70.2% at 48 h treatment at a dose of 3.125 μg/ml and there was no significant change in the viability has the dose increased from 6.25 to 25 μg/ml (66.5 and 67.9%, respectively) and the cell viability slightly dropped from 50 μg/ml to 57.8%. Interestingly, the lowest cell viability was noticed at 48 h treatment. For TQ-ACNP, the cell viability of 3T3 cells decreased slightly has the dose increased from 3.125 to 50 μg/ml at all treatment period. At 24 h the viability of TQ-ACNP decreased from 100 to 74.6%, 99–81.1% (48 h), and 72.1–68.3% (72 h), respectively. Dox/TQ-ACNP also showed slight decreased in the cell viability of 3T3 cells as the dose and treatment time increased. At 24 h the viability of Dox/TQ-ACNP decreased from 96.9–71.7%, 96.1–59.7% (48 h), and 94.7–65.2% (72 h) for 3.125 and 50 μg/ml doses, respectively.

**Figure 3:**
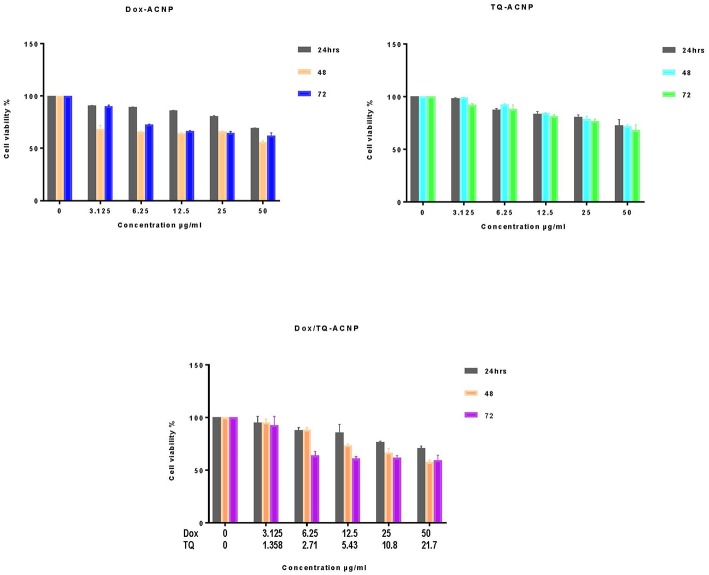
Percentage cell viability of MCF10A cells after treatments with drug loaded ACNP at concentration ranging from 0 to 50 μg/ml for 24, 48, and 72 h.

Figure 3 shows the cell viability of MCF-10A after treatment with Dox-ACNP, TQ-ACNP, and Dox/TQ-ACNP for 24, 48, and 72 h. For Dox-ACNP, the 24 h the cell viability of MCF-10A decreased from 90.7 to 69.4% at treatment dose of 3.125 and 50 μg/ml, respectively. MCF-10A viability decreased from 68.2% at 48 h treatment at a dose of 3.125 μg/ml to 55.8% at dose 50 μg/ml. Cell viability decreased from 90.1 to 62.1% at 78 h. For TQ-ACNP, the cell viability of MCF-10A cells progressively decreased as the dose increased from 3.125 to 50 μg/ml at all treatment period. At 24 h the viability decreased from 98.3 to 72.6%, 99 to 72.1% (48 h), and 92.1 to 68.3% (72 h), respectively. Dox/TQ-ACNP also showed a progressive decreased in the cell viability as the dose and treatment time increased. At 24 h the cell viability decreased from 94.9 to 70.4%, 95.6 to 57.8% (48 h), and 92.4 to 59% (72 h) for 3.125 and 50 μg/ml doses, respectively.

At all treatment period the cell viability of Dox-ACNP, TQ-ACNP, and Dox/TQ-ACNP in both 3T3 and MCF-10A were more than 50%. This shows that drug-loaded ACNP is relatively safe in non-neoplastic cells. Kulsharova et al. (25) reported that Dox-loaded Gelatin nanoparticles coated with Cathepsin D-specific peptides b did not cause any cytotoxicity to 3T3 fibroblast cells, and increased in cell proliferation was noticed. They attributed this finding to the peptide coating of the nanoparticle which is specific to biomarker associated with cancer cells. Sahu et al. (26) also showed an increased in cell viability of NIH 3T3 cells when treated with paclitaxel-loaded nanoparticles as compared to HeLa cells.

### Safety Assessment of Drug-Loaded ACNP in Non-neoplastic Cells

**Figure 4:**
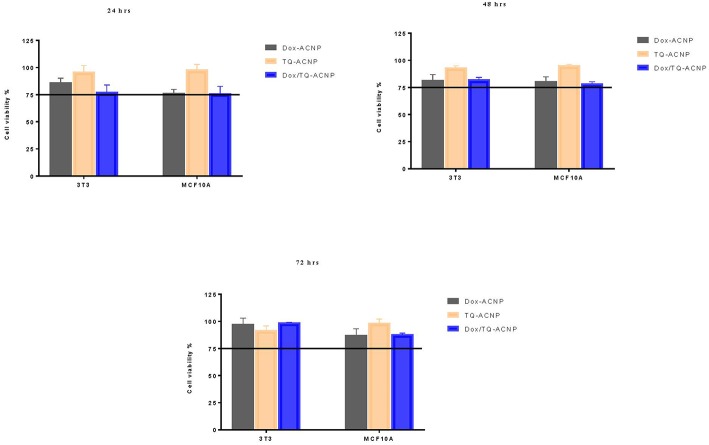
Graphical representation of the percentage viability of 3T3 and MCF10A at IC_50_ values of MDA MB 231 cells.

Most anticancer drugs are cytotoxic to the normal cells. To further ascertain the safety of Dox-ACNP, TQ-ACNP and Dox/TQ-ACNP in normal cells, we tested their IC_50_ dosage in MDA-MB-231 breast cancer cell line (Table 1) on MCF-10A and 3T3 non-neoplastic cell lines. The cell viability >75% was considered non-cytotoxic to the non-neoplastic cells (27). It was observed that the IC_50_ dosage of Dox-ACNP, TQ-ACNP, and Dox/TQ- at 24, 48, and 72 h, respectively, which induced 50% cell viability reduction in of MDA-MB-231 neoplastic cells were not toxic to MCF-10A and 3T3 non-neoplastic cells at all period of treatment (Figure 4). As shown in Figure 4, the cell viability of 3T3 and MCF-10A after treatment with neoplastic IC_50_ value of Dox-ACNP, TQ-ACNP, and Dox/TQ-ACNP at 24 h was 86.3, 96.3, 77.4% and 76.5, 98.1, 76.3%, respectively. At 48 h the percentage viability was 81.6, 93.3, 82.3%, and 80.7, 95.2, 78.4% for 3T3 and MCF10A, respectively. At 72 h the percentage viability for 3T3 and MCF10A 97.4, 92, 98.9% and 87.4, 98.7, 88.2% after treatment with Dox-ACNP, TQ-ACNP, and Dox/TQ-ACNP, respectively.

### Morphology Assessment

#### Light Microscopy Cell Imaging

**Figure 5:**
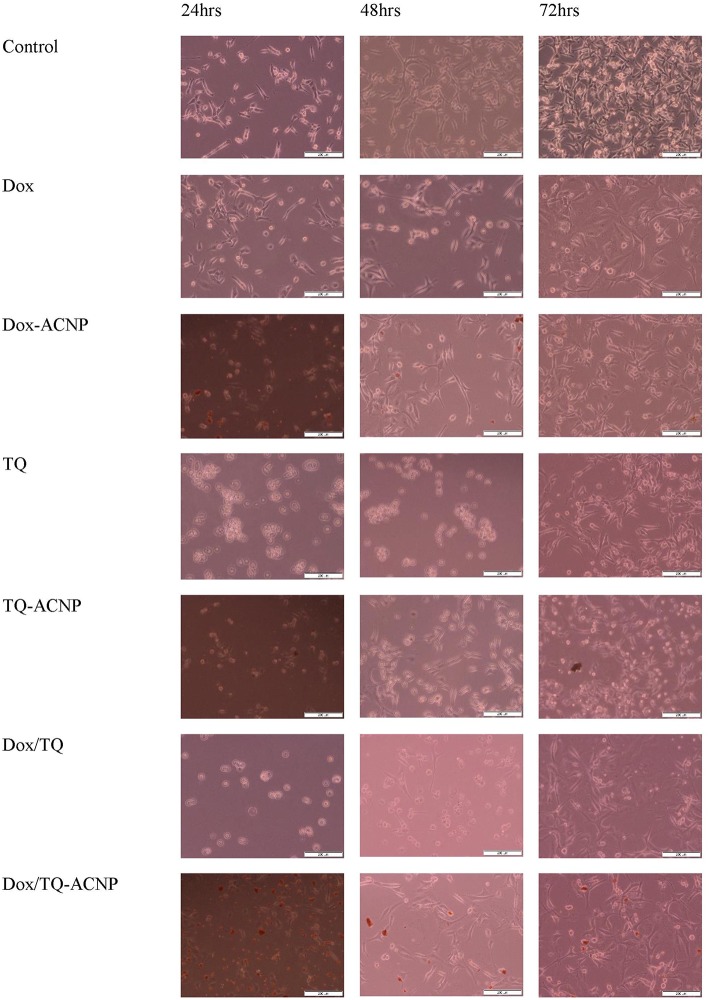
Inverted light microscopy images of MDA MB 231 cells treated at 24, 48, and 72 h.

MDA-MB-231 breast cancer cell line was treated with free and drug-loaded ACNP for 24, 48, and 72 h. The morphological observations in control revealed adherent growth with increased in cell population as the culture day increases. Decrease in cell population for all treatment was observed compared to control but it was more obvious in the Dox/TQ-ACNP treated group at 24, 48, and 72 h of treatment period. In addition, cell rounding and detachment was observed in all the treated groups, most prominent in TQ treated group at 24 and 48 h (Figure 5). The small reddish/dark dots observed in drug-loaded ACNPs are aggregated nanoparticles. The decreased in cell population, cell rounding and detachment demonstrate the occurrence of apoptosis.

#### Scanning Electron Microscopy (SEM)

**Figure 6:**
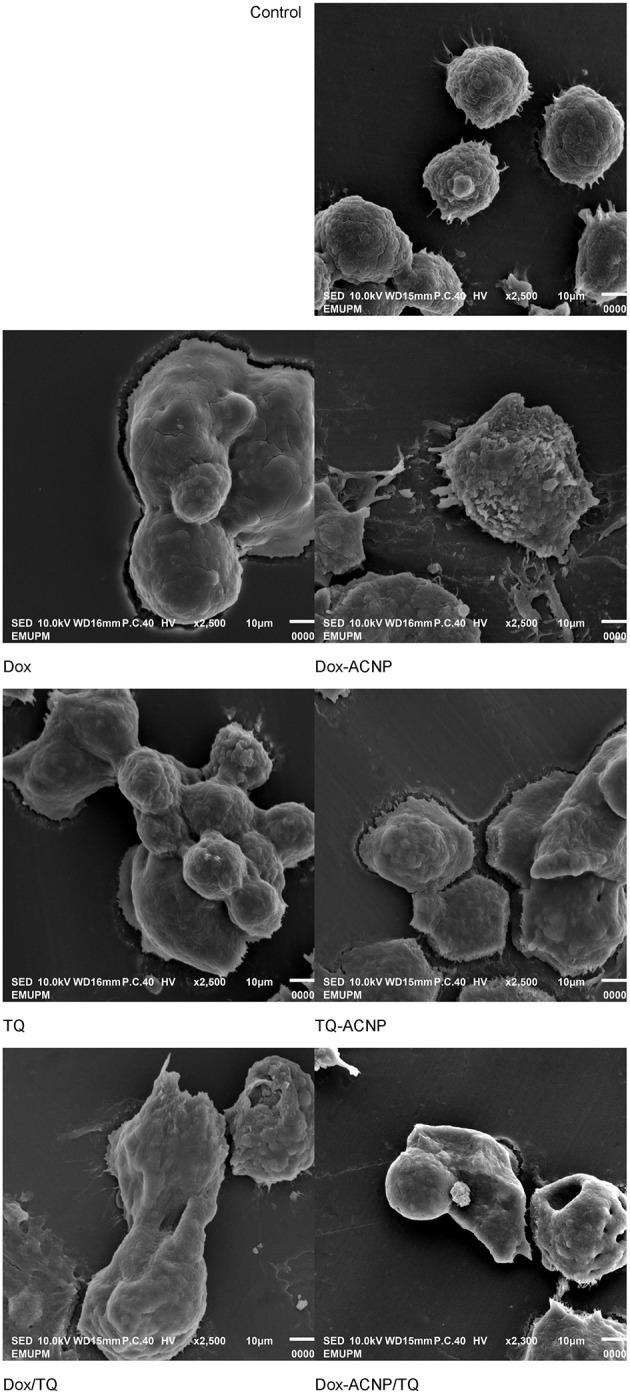
Scanning electron micrograph of untreated, and treated MDA MB 231 cells at 72 h of treatment (drug doses: Dox-ACNP and Dox 0.7 μg/ml, TQ-ACNP, and TQ 0.9 μg/ml, Dox/TQ-ACNP and DoxTQ 0.2 μg/ml).

The surface morphological changes of MDA-MB-231 cells was observed by SEM as shown in Figure 6. For control, the round cells with smooth surface and normal cell membrane was observed. The morphological changes observed in the treated groups include membrane blebbing, cell shrinkage and apoptotic bodies (cell fragments). Loss of cell membrane integrity was only observed in Dox/TQ-ACNP treated group. Membrane blebbing, cell shrinkage and apoptotic bodies are features of apoptosis while loss of cell membrane integrity is a feature of necrosis. It should be noted that the dose of 0.2 μg/ml was used to compare the morphological changes between Dox/TQ-ACNP and free Dox/TQ combination at 72 h of treatment. This dose is approximately two times more than IC_50_ of Dox/TQ-ACNP (0.095 μg/ml, Table 1). 0.2 μg/ml is within the range of cytotoxic dose for Dox/TQ-ACNP.

#### Transmission Electron Microscopy (TEM)

**Figure 7:**
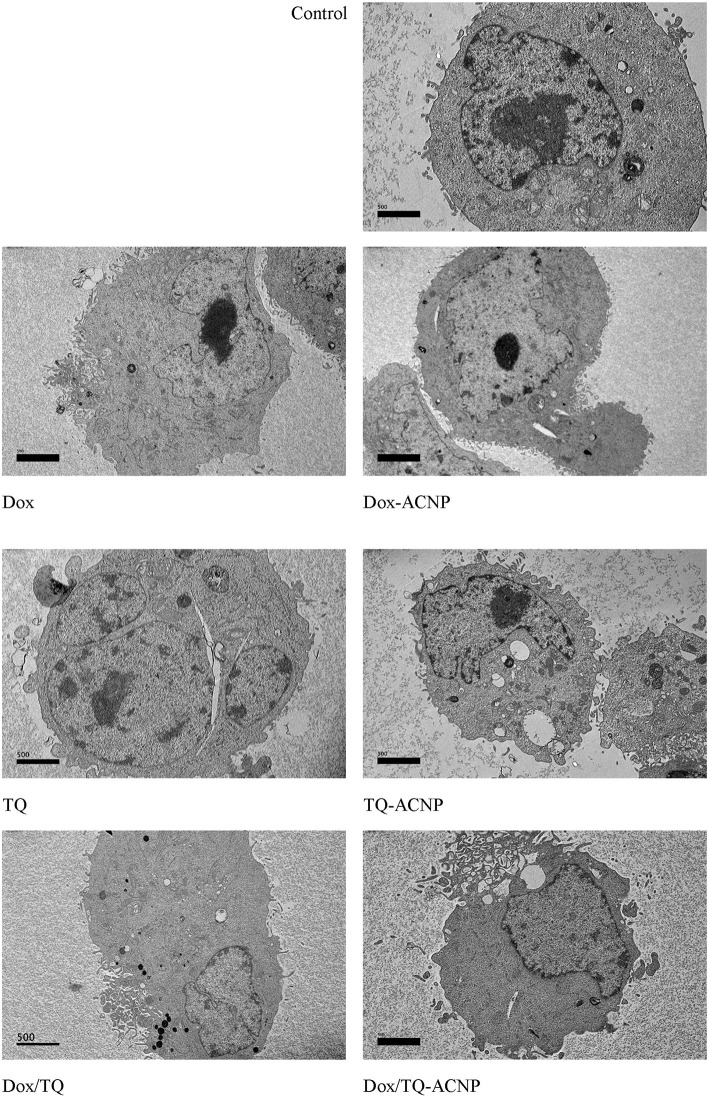
Micrograph showing transmission electron microscopy of untreated, and treated MDA MB 231 cells at 72 h of treatment (drug doses: Dox-ACNP and Dox 0.7 μg/ml, TQ-ACNP, and TQ 0.9 μg/ml, Dox/TQ-ACNP and DoxTQ 0.2 μg/ml).

TEM micrographs showing ultrastructure of MDA-MB-231 cells (Figure 7). The control showed intact cell membrane, normal cell organelles, intact nuclear membrane, nucleolus and well distributed chromatin materials. The ultrastructural changes noticed in the treatment groups include cell shrinkage and membrane blebbing. Little to no changes was seen in the cell organelles. Only Dox/TQ-ACNP treated group showed disruption of the cell membrane which is an indication of necrosis, this is consistent with what was observed in SEM. This results show that the combination of TQ/Dox-loaded ACNP could effectively kill breast cancer cells at a much lower dose than its free counterpart or the single drug-loaded ACNP.

### Apoptosis

**Figure 8:**
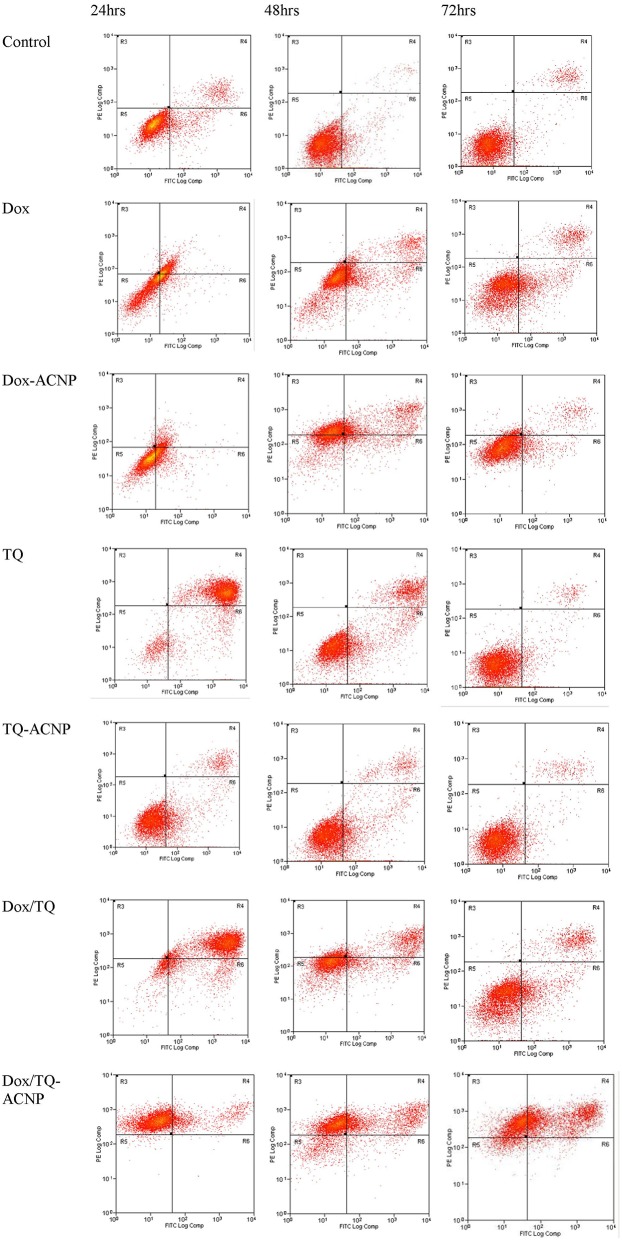
Flow cytometry results of Annexin V assay showing different distribution of cell cytopathology in untreated and treated MDA MB 231 at 24, 48, and 72 h.

Phosphatidylserine protein is normally found in the inner cell membrane and has a high affinity for Annexin V. during the process of apoptosis, phosphatidylserine protein is exposed to the cell surface. Therefore, Annexin V with fluorescein will bind to the exposed phosphatidylserine and will be detected by flow cytometer for apoptosis measurement. PI enters the cells through the damaged cell membrane and bind with DNA of necrotic cells. Figure 8 shows Annexin V/PI assay results in MDA-MB-231 cells using flow cytometry. It showed different distribution of the cytopathology i.e., early apoptosis, late apoptosis and necrosis in control and treatment groups at 24, 48, and 72 h. For free Dox treatment group, the percentages of early apoptotic cells at 24, 48, and 72 h were 18.6, 20, and 19%, respectively, while for Dox-ACNP were 22.8, 7.67, and 7.06%, respectively. The percentages of late apoptotic cells for free Dox at 24, 48, and 72 h were 24.4, 10.9, and 8.44%, respectively, while for Dox-ACNP were 8.47, 23, and 5.1%, respectively. The percentage of necrotic cells for free Dox at 24, 48, and 72 h were 1.7, 0.88, and 0.34%, respectively, while for Dox-ACNP were 1.04, 40.8, and 4.1%, respectively. Dose of 2.1 μg/ml, 0.6 and 0.7 μg/ml at 24, 48, and 72 h treatment, respectively, were used to compare apoptotic effect of free Dox and Dox-ACNP. At 48 h, Dox-ACNP was more effective in inducing apoptosis than free Dox. At 24 and 72 h there was no significant difference in the induction of apoptosis in both treatments.

A dose of approximately 1 μg/ml was used to compare the induction of apoptosis between free TQ and TQ-ACNP at 24, 48, and 72 h of treatment. For TQ-ACNP the percentages of early apoptotic cells were 19.28, 20.8, and 3.06%, respectively, while for TQ were 9.19, 18.9, and 5.1%, respectively. The percentages of late apoptotic cells for TQ-ACNP at 24, 48, and 72 h of treatment were 6.4, 6.9, and 3.3% respectively, while for TQ were 82.5, 19.6, and 2.4%, respectively. The percentage of necrotic cells for both TQ and TQ-ACNP were <1% at all time periods. TQ was more effective in inducing apoptosis, only at 24 h, when compared with TQ-ACNP in a time dependent manner. It seems the duration of action of TQ is short and a daily dosing will be more preferred. There is no significant different between TQ-ACNP and control at all time periods. This may be due to the delayed release of loaded TQ from TQ-ACNP; drug release from ACNP at normal pH is rather slow.

For Dox/TQ-ACNP, the percentages of early apoptotic cells at 24, 48, and 72 h of treatment were 0.12, 2.7, and 2.5% respectively, while for Dox/TQ were 12.3, 10.6, and 15%, respectively. The percentages of late apoptotic cells at 24, 48, and 72 h of treatment for Dox/TQ-ACNP were 13.3, 29.8, and 43.1%, respectively, while for Dox/TQ were 76.9, 18, and 10%, respectively. The percentage of necrotic cells at 24, 48, and 72 h of treatment for Dox/TQ-ACNP were 85.5, 55.8, and 44.9%, respectively, while for Dox/TQ were 1.4, 13, and 0.8%, respectively. Dox/TQ shared the same cytopathologic features of both free Dox and free TQ as shown in **Figure 10**; high percentage of late apoptotic cells at 24 h with progressive increase in viable cells at 48 and 72 h. The cytopathologic features in Dox/TQ-ACNP was relatively consistent from 24 h through 72 h of treatment. This may be due to the gradual and continuous release of Dox and TQ from ACNP, and the synergism between Dox and TQ. The flow cytometry cytopathologic feature seen with Dox/TQ-ACNP is consistent with the morphological findings. This result showed that the combined drug-loaded ACNP showed the most cytopathologic features in treated breast cancer cells when compared to the single drug-loaded ACNP or free drugs.

### Cell Cycle Analysis

**Figure 9:**
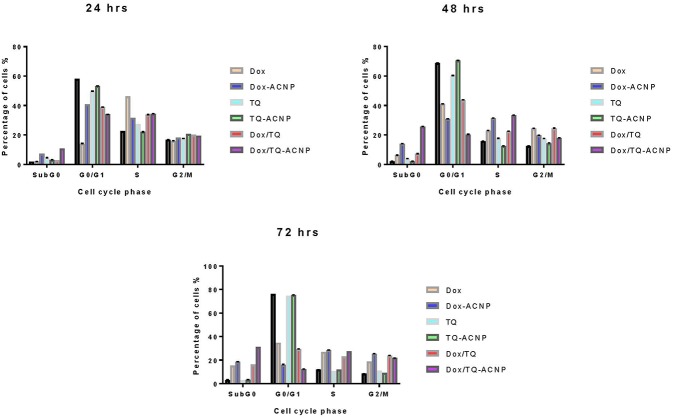
Graphical representation of effect of various treatment on the cell cycle distribution of MDA MB 231 cells.

To assess the influence of free and drug-loaded ACNP on the cell cycle distribution of breast cancer cells, MDA-MB-231 cells were treated with various treatments under investigation for 24, 48, and 72 h (Figure 9). No treatment causes significant anti-proliferative effect by increasing the cell population at G0/G1 phase after the three treatment period. Dox (14%) had the least effect on G0/G1 phase at 24 h while Dox/TQ-ACNP had the least effect at 48 and 72 h (20.2 and 12.4%, respectively).

All of the treatment, except TQ-ACNP at 72 h of treatment, cause significant S-phase arrest by increasing its cell population when compared to control. Dox (46.2%) had the most cell population in S phase at 24 h of treatment while Dox/TQ-ACNP had the most cell population in S phase at 48 and 72 h of treatment (33.3 and 27.6% increase in cell population, respectively).

TQ-ACNP had the least effect at all treatment time (22.1, 12.5, and 11.9%, respectively). The number of cells at the G2/M phase was increased in Dox and Dox/TQ treatment (24.3 and 24.4%, respectively) at 48 h and, Dox-ACNP, Dox/TQ and Dox/TQ-ACNP at 72 h in descending order (25.4, 23.9, and 21.8%, respectively), indicating cycle arrest at G2/M phase.

SubG0 represents cellular DNA content <2N. This indicates nuclear fragmentation and it is a characteristic of apoptosis. After 24 h, all the treatment groups except Dox induced significant increase in SubG0 cell population. Only TQ-ACNP at 48 h and TQ-ACNP and TQ at 72 h did not significantly increased in SubG0 cell population. Dox/TQ-ACNP has the most percentage of in SubG0 i.e., dead cells. This is consistent with the findings in Annexin V and morphological assay.

### Wound Healing and Cell Invasion Assay

#### Scratch Assay

**Figure 10:**
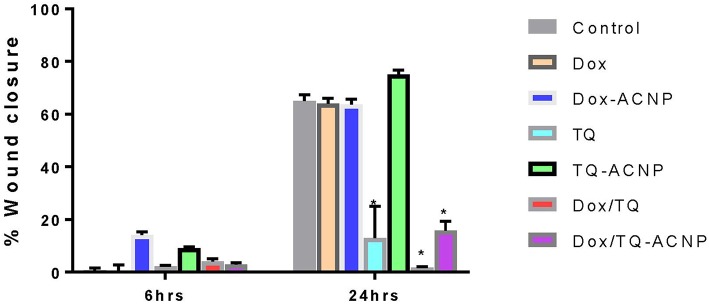
Graphical representation of wound closure in control and treated MDA MB 231 cells at 6 and 24 h post scratching. ^*^ Represents *P* < 0.05 compared to control.

Also known as wound healing assay, is a low cost, easy to do assay that is commonly used to study the movement of cells. The wound closure in this assay is as a result of both cell migration and cell proliferation. The graphical representation of wound healing assay after 6 and 24 h in control and treated samples is shown in Figure 10. The percentage of wound closure was highest in the TQ-ACNP treatment group (75%) when compared to other treatment groups at 24 h. In ascending order, Dox/TQ (1.7%), TQ (13%), Dox/TQ-ACNP (15.9%) had the least closure at 24 h. Dox/TQ and TQ are free drugs and their onset of action is quicker than Dox/TQ-ACNP in which the encapsulated drugs have to be release from ACNP before they can exert their effect. This may be the reason why Dox/TQ and TQ reduced cell migration more effectively compared to Dox/TQ-ACNP. Antiproliferative effect of these treatment also contributed to the impaired cell migration seen.

#### Invasion Assay

**Figure 11:**
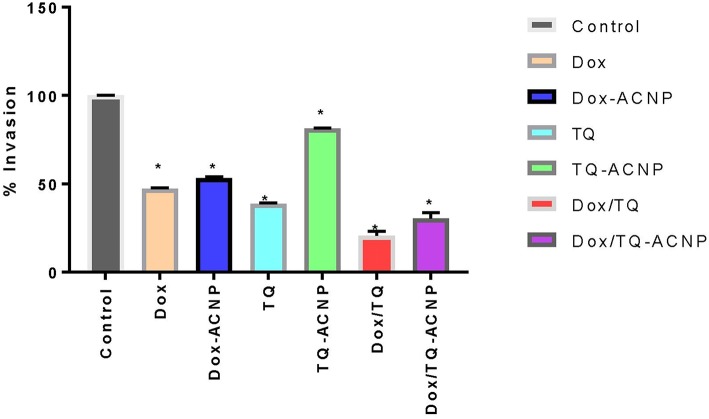
Graphical representation of percentage cell invasion across basement membrane compared to control. ^*^ Represents *P* < 0.05 compared to control.

The invasiveness of MDA-MB-231 breast cancer cells was examined with a commercial cell invasion assay kit. The percentage of cell invasion across basement membrane was shown in Figure 11. Dox/TQ (20.5%) had the most effect on cell invasion followed by Dox/TQ-ACNP (30.4%) and TQ (38.7%). TQ-ACNP (81.4 %) had the least effect on invasion of cell across the basement membrane, followed by Dox-ACNP (53.2%) and Dox (47.3%) of cells invaded the membrane when compared to control.

From the results of wound healing and cell migration assay, it can be seen that TQ played a major role in inhibition of breast cancer metastasis; but the combination of Dox and TQ was more effective. Dox/TQ-ACNP has the most anti-metastatic effect when compared to Dox-ACNP and TQ-ACNP. Thymoquinone has been shown to inhibit cancer cell migration and invasion. TQ suppressed MDA-MB-231 breast cancer cell migration and multiple organ metastases (lung, brain, and bone) in metastasis breast tumor mouse model. The proposed mechanism of anti-metastatic effects in breast cancer was by down regulation of NF-κB regulated CXCR4 expression (28). TQ also controlled melanoma metastasis (29), inhibit cell migration and invasion in cervical cancer cells (30); human glioma cells by down regulating focal adhesion kinase and inhibiting secretion of matrix metalloproteinase (31).

## Conclusion

The combination therapy showed enhanced apoptosis, reduction in cellular migration and invasion when compared to the single drug-loaded CaCO_3_ nanoparticle and the free drugs. The results from this study showed that the combined drug-loaded cockle shell-derived aragonite calcium carbonate nanoparticles showed higher efficacy in breast cancer cells at lower dose of doxorubicin or thymoquinone. Enhanced cellular uptake, nuclear localization of Dox/TQ-ACNP, gradual and continuous release, pH sensitive release that prompt quick release of drugs in late endosome/lysosome, as well as the synergistic interaction between Dox and TQ contributed to the increased efficacy of Dox/TQ- ACNP.

## Data Availability

All datasets generated for this study are included in the manuscript and/or the supplementary files.

## Author Contributions

KI and AZ contributed to the design of the study, acquisition, analysis and interpretation of data, writing, and reading of the manuscript. NN and MA assisted in interpretation of data and reading of the manuscript. AZ provided the final approval of the manuscript for publication.

### Conflict of Interest Statement

The authors declare that the research was conducted in the absence of any commercial or financial relationships that could be construed as a potential conflict of interest.
